# Optimised graphite/carbon black loading of recycled PLA for the production of low-cost conductive filament and its application to the detection of *β*-estradiol in environmental samples

**DOI:** 10.1007/s00604-024-06445-7

**Published:** 2024-06-07

**Authors:** Karen K. L. Augusto, Robert D. Crapnell, Elena Bernalte, Sabri Zighed, Anbuchselvan Ehamparanathan, Jessica L. Pimlott, Hayley G. Andrews, Matthew J. Whittingham, Samuel J. Rowley-Neale, Orlando Fatibello-Filho, Craig E. Banks

**Affiliations:** 1 Faculty of Science and Engineering, Manchester Metropolitan University, Chester Street, M1 5GD Manchester, Great Britain; 2 Laboratório de Analítica, Bioanalítica, Biosensores, Electroanalítica e Sensores, Departamento de Química, Universidade Federal de São Carlos (UFSCar), Sao Carlos, CP 676, 13560-970 SP Brazil; 3 Department of Physical Measurements, Sorbonne Paris North University, Place du 8 Mai 1945, Saint-Denis, 93200 France

**Keywords:** Additive manufacturing, 3D-printing, Electroanalysis, Square wave voltammetry, Environmental analysis, *β*-Estradiol

## Abstract

The production, optimisation, physicochemical, and electroanalytical characterisation of a low-cost electrically conductive additive manufacturing filament made with recycled poly(lactic acid) (rPLA), castor oil, carbon black, and graphite (CB-G/PLA) is reported. Through optimising the carbon black and graphite loading, the best ratio for conductivity, low material cost, and printability was found to be 60% carbon black to 40% graphite. The maximum composition within the rPLA with 10 wt% castor oil was found to be an overall nanocarbon loading of 35 wt% which produced a price of less than £0.01 per electrode whilst still offering excellent low-temperature flexibility and reproducible printing. The additive manufactured electrodes produced from this filament offered excellent electrochemical performance, with a heterogeneous electron (charge) transfer rate constant, *k*^0^ calculated to be (2.6 ± 0.1) × 10^−3^ cm s^−1^ compared to (0.46 ± 0.03) × 10^−3^ cm s^−1^ for the commercial PLA benchmark. The additive manufactured electrodes were applied to the determination of *β*-estradiol, achieving a sensitivity of 400 nA µM^−1^, a limit of quantification of 70 nM, and a limit of detection of 21 nM, which compared excellently to other reports in the literature. The system was then applied to the detection of *ß*-estradiol within four real water samples, including tap, bottled, river, and lake water, where recoveries between 95 and 109% were obtained. Due to the ability to create high-performance filament at a low material cost (£0.06 per gram) and through the use of more sustainable materials such as recycled polymers, bio-based plasticisers, and naturally occurring graphite, additive manufacturing will have a permanent place within the electroanalysis arsenal in the future.

**Graphical abstract:**

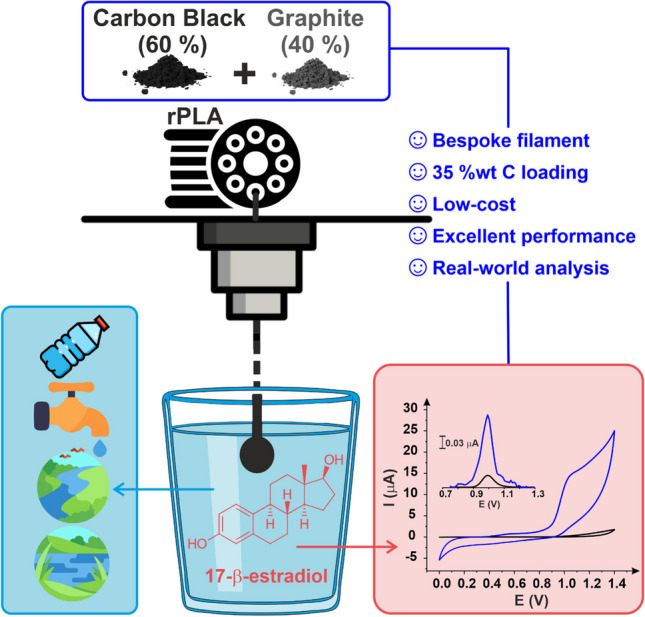

**Supplementary Information:**

The online version contains supplementary material available at 10.1007/s00604-024-06445-7.

## Introduction

The 17 sustainable development goals developed by the United Nations are an urgent call for action in a global partnership between developed and developing countries [1]. They form the core of the 2030 Agenda for Sustainable Development, which was adopted by all UN member states in 2015, and cover a vast range of challenges that are crucial to overcome for the prosperity of ourselves and our planet. Specifically, sensing devices play a vital role to ensuring that many of these goals are met and then subsequently monitored with accuracy and reliability. In particular, Goals 3 and 6, Good Health and Wellbeing and Clean Water and Sanitation, respectively, require the constant monitoring of water supplies to guarantee that standards are met in all areas. Water pollution is a huge issue worldwide, with freshwater accounting for a tiny proportion of the world’s total water supply. Both natural and anthropogenic activities can cause changes in the water quality, with the latter being mainly caused by agricultural processes, inefficient irrigation practices, deforestation, domestic sewage, mining, and industrial effluents [2]. One compound seen increasingly within water sources is *β*-estradiol (or 17-*β*-estradiol), a steroidal hormone important within female reproductive processes, regulating bone growth, and brain maintenance [3]. This hormone is excreted by humans in urine and released by pharmaceutical industries, whereby it bioaccumulates in water sources and can lead to significant complications for fauna in the local ecosystem [4–6]. It is such the importance of the known environmental risk caused by the presence of estrogens in water ecosystems [7] that, from January 2023, the European Commission has included *β*-estradiol in the first watch list of substances of concern for water intended for human consumption [8, 9].

Monitoring of these environmental contaminants, such as *β*-estradiol, is commonly carried out in a laboratory utilising standard benchtop techniques such as high performance liquid chromatography coupled with tandem quadrupole mass spectrometry (HPLC-MS/MS) [10, 11] or liquid chromatography-mass spectrometry (LC-MS) [12, 13]. These testing regimes are expensive, slow, require skilled operators to perform meticulous preparation, and involve the transportation of samples from the environment to the lab. All these factors contribute to increase the ineffectiveness of environmental monitoring, with costs either being too high or results being unreliable due to timescales. However, the use of modern electrochemical devices for the analysis of environmental contaminants has the potential to remove these roadblocks, allowing rapid quantification of trace-level pollutants directly in the field and minimising the risk associated with the sampling, storage, and transportation of environmental specimens. Nowadays, portable and miniaturised potentiostats are readily available for less than £1000, do not require highly skilled end-users, and allow for the collection of large amounts of real-time data in decentralised settings. For some time, screen-printed electrodes have been the only alternative electrochemical technology for transitioning analysis from the laboratory into the field [14, 15]. These electrodes utilise the deposition of conductive ink onto a flat ceramic or plastic substrate to produce typically single-use electrodes which can easily be used in situ with no additional preparation. Although these electrodes have excellent scales of economy and are simple to use, their disposable nature and the materials typically used in their fabrication make them a questionable option environmentally.

One area of interest for portable sensor development is additive manufacturing, which utilises the deposition of material in a layer-by-layer fashion to produce a 3D object from a computer-aided design file. This technology allows for on-demand manufacturing, low (potentially zero) waste, short lead times, low costs for short production runs, a high degree of customisability, ease of global collaboration, and the ability to produce complex parts [16]. Within the field of electrochemistry, fused filament fabrication (FFF) has become extremely popular due to its inexpensive entry level, availability of commercial electrically conductive filament, and reproducible results. As such, it has been utilised throughout electrochemical research labs for the production of accessories, specialist equipment, electrochemical cells, and, of course, electrodes [17, 18]. Through the use of commercially available conductive filament, many electroanalytical sensors have been reported for the determination of environmental contaminants such as pesticides [19], pharmaceuticals [20, 21], and heavy metals [22, 23]. Due to the flexibility of FFF and the available printers, full electrochemical platforms have been produced in a single print, with any number of electrodes embedded within the cell [19, 24]. Although these sensing platforms offer unique advantages, their electroanalytical performance remains substandard compared to traditional electrodes, primarily due to the limited conductivity of the filament.

To overcome this issue, researchers have begun to produce their own bespoke filaments with improved conductivities. This is typically achieved through mixing nanocarbons with the base polymer through either solvent or thermal mixing, with thermal being the preferred choice in terms of production timescales, removal of solvent requirements, and the possibility to power machinery through green energy sources [25]. Various bespoke filaments have now been reported for use within electroanalysis, such as for the determination of bisphenol A in water samples [26]. To further improve bespoke conductive filaments, researchers have experimented with novel combinations of multi-walled carbon nanotubes with carbon black to produce high-performance conductive filament [27] or mixing graphite with carbon black to reduce the cost and improve the sustainability of the filament [28]. This work looks to build upon this work through understanding the optimal carbon black and graphite composition whilst also maximising the loading to achieve the best possible electrode at the lowest cost. Reducing the cost of electrode production is a significant step toward the commercial application of this technology, but the improvements in sustainability are vital to keep this field in line with the UN’s sustainable development goals, such as Goal 12: Sustainable Consumption and Production.

In addition to the incorporation of graphite, mentioned above, researchers have taken significant steps toward improving the overall sustainability of additive manufacturing electrochemistry [29]. Sigley and co-workers described the first use of recycled poly(lactic acid) (PLA) from coffee pods to produce both conductive and non-conductive filaments for the development of an electroanalytical sensor applied for caffeine detection [30]. Crapnell and co-workers then took this approach further through the replacement of the poly(ethylene succinate) plasticiser with the bio-based reagent castor oil [26], whilst also showing the effectiveness of recycling the used electroanalytical sensing platforms into a new filament that can once again print the same electroanalytical platforms whilst maintaining the performance of the original material [31].

As such, in this work, we look to optimise the production of electrically conductive additive manufacturing filament for excellent electrochemical performance, low production cost, and improved sustainability. We aim to achieve this through optimising the graphite-to-carbon black ratio in the filament whilst also using recycled PLA and the bio-based plasticiser castor oil. Once the nanocarbon ratio is optimised, we look to incorporate the highest loading possible to produce the best-performing conductive filament and then utilise this for the detection of *β*-estradiol within different water sources.

## Experimental section

### Chemicals

All chemicals used throughout this work were used as received without any further purification. All aqueous solutions were prepared with deionised water of a measured resistivity not less than 18.2 MΩ cm, sourced from a Milli-Q Integral 3 system from Millipore UK (Watford, UK). Hexaammineruthenium(III) chloride (> 98%), castor oil, potassium ferricyanide (99%), potassium ferrocyanide (98.5–102%), sodium hydroxide (> 98%), potassium chloride (99.0–100.5%), *β*-estradiol (≥ 98%), graphite powder (< 20 μm), sulfuric acid (99.999%), and methanol (≥ 99.9%) were purchased from Merck (Gillingham, UK). Carbon black was purchased from PI-KEM (Tamworth, UK). Recycled poly(lactic acid) (rPLA) was purchased from Gianeco (Turin, Italy). Commercial conductive PLA/carbon black filament (1.75 mm, ProtoPasta, Vancouver, Canada) was purchased from Farnell (Leeds, UK). River water samples were obtained in accordance with EPA guidelines from the River Irwell, Greater Manchester, UK (approx. location: 53.517464, −2.302739). Lake water samples were obtained in accordance with EPA guidelines from Drinkwater Park, Greater Manchester, UK (approx. location: 53.519601, −2.298174). Tap water samples were obtained from laboratory 5.39, John Dalton Tower, Manchester, UK. Bottled water samples were Highland Spring Still Water (500 mL) obtained from a local convenience store.

### Recycled filament production

All rPLA was dried in an oven at 60 °C for a minimum of 2.5 h before use to remove any residual water in the polymer. The polymer compositions were prepared through the addition of appropriate amounts of rPLA, castor oil, CB, and graphite in a chamber of 63 cm^3^. All filaments made throughout this work utilised 10 wt% castor oil as a plasticiser [26], and the amounts of PLA, CB, and graphite were altered and are labelled as such throughout the manuscript. The compounds were mixed using a Thermo Haake Polydrive dynameter fitted with a Thermo Haake Rheomix 600 (Thermo-Haake, Germany) at 190 °C with Banbury rotors at 70 rpm for 5 min. The resulting polymer composites were allowed to cool to room temperature before being granulated to create a finer particle size using a Rapid Granulator 1528 (Rapid, Sweden). The polymer composites were collected and processed through the hopper of a EX6 extrusion line (Filabot, VA, USA). The EX6 was set up with a single screw with four set heat zones of 60, 190, 195, and 195 °C, respectively. The molten polymer was extruded from a 1.75-mm die head, pulled along an Airpath cooling line (Filabot, VA, USA), and collected on a spool. After which, the filament was then ready to use for additive manufacturing (AM).

### Additive manufacturing of the electrodes

All computer designs and 3MF files in this manuscript were produced using Fusion 360® (Autodesk®, CA, USA). These files were sliced and converted to GCODE files in PrusaSlicer (Prusa Research, Prague, Czech Republic). The additive manufactured electrodes were produced using fused filament fabrication (FFF) technology on a Prusa i3 MK3S+ (Prusa Research, Prague, Czech Republic). All additive manufactured electrodes were printed onto a standard polyetherimide (PEI) print bed using identical printing parameters, namely, a 0.6 mm nozzle with a nozzle temperature of 215 °C, 100% rectilinear infill [32], 0.1 mm layer height, and a print speed of 35 mm s^−1^.

### Physicochemical characterisation

X-ray photoelectron spectroscopy (XPS) data were acquired using an AXIS Supra (Kratos, UK), equipped with a monochromatic Al X-ray source (1486.6 eV) operating at 225 W and a hemispherical sector analyser. It was operated in fixed transmission mode with a pass energy of 160 eV for survey scans and 20 eV for region scans, with the collimator operating in slot mode for an analysis area of approximately 700 × 300 μm, and the FWHM of the Ag 3d5/2 peak using a pass energy of 20 eV was 0.613 eV. The binding energy scale was calibrated by setting the graphitic sp^2^ C 1s peak to 284.5 eV; this calibration is acknowledged to be flawed [33] but was nonetheless used in the absence of reasonable alternatives and because only limited information was to be inferred from absolute peak positions.

Scanning electron microscopy (SEM) micrographs were obtained using a Crossbeam 350 Focussed Ion Beam – scanning electron microscope (FIB-SEM) (Carl Zeiss Ltd., Cambridge, UK) fitted with a field emission electron gun. Secondary electron imaging was completed using a secondary electron secondary ion (SESI) detector. Samples were mounted on the aluminium SEM pin stubs (12 mm diameter, Agar Scientific, Essex, UK) using adhesive carbon tabs (12 mm diameter, Agar Scientific, Essex, UK) and coated with a 5 nm layer of Au/Pd metal using a Leica EM ACE200 coating system before imaging.

Raman spectroscopy was performed on a DXR Raman Microscope (Thermo Scientific Inc., Waltham, MA, USA) configured with a 532 nm laser and operated using OMNIC 9 software.

### Electrochemical experiments

All electrochemical experiments were performed on an Autolab 100 N potentiostat controlled by NOVA 2.1.7 (Utrecht, The Netherlands). Identical additive manufactured electrodes were used throughout this work for all filaments, printed in a lollipop shape (Ø 5 mm disc with 8 mm connection length, 2 mm width, and 1 mm thickness [34]) alongside an external commercial Ag|AgCl/KCl (3 M) reference electrode with a nichrome wire counter electrode. All solutions of [Ru(NH_3_)_6_]^3+^ were purged of O_2_ thoroughly using N_2_ prior to any electrochemical experiments. Solutions of [Fe(CN)_6_]^4−/3−^ were prepared in the same way without the need of further degassing. Baseline correction in square wave voltammograms was performed using the standard ‘moving average’ method included in the NOVA 2.1.7 software.

Electrochemical impedance spectroscopy (EIS) was recorded in the frequency range of 0.1 Hz to 100 kHz, applying 10 mV of signal amplitude to perturb the system under quiescent conditions. NOVA 2.1.7 software was used to fit the Nyquist plots obtained to an adequate equivalent circuit.

The activation of the additive manufactured electrodes was performed before all electrochemical experiments. This was achieved electrochemically in NaOH (0.5 M), as described in the literature [35]. Briefly, the additive manufactured electrodes were connected as the working electrode in conjunction with a nichrome wire coil counter and Ag|AgCl/KCl (3 M) reference electrode and placed in a solution of 0.5 M NaOH. Chronoamperometry was used to activate the additive manufactured electrodes by applying a set voltage of + 1.4 V for 200 s, followed by applying − 1.0 V for 200 s. The additive manufactured electrodes were then thoroughly rinsed with deionised water and dried under compressed air before further use.

## Results and discussion

### Optimisation of graphite and carbon black loading

To optimise the ratio of CB to graphite within the recycled PLA (rPLA), different compositions were made at a total nanocarbon loading of 15 wt%. The overall loading was reduced compared to previous work [28] to allow for easier differentiation between the measured resistances; as with high loadings, all filaments tend to perform well. Filaments were made with CB to graphite ratios of 100:0, 80:20, 60:40, 40:60, and 20:80, but not 0:100 because of the high degradation of the rPLA upon mixing with 100% graphite due to its abrasive nature. We note that high levels of only graphite loading have been reported using solvent mixing methods [36, 37]; however, thermal is the preferred method used here for reasons outlined in the introduction. All the filaments that were produced had excellent low-temperature flexibility, as shown in Figure S1. Following this, the resistance across 10 cm of filament was measured, and this allowed for comparisons between the filaments but also to the commercially available conductive PLA, as this is a quoted value of 2–3 kΩ for a 1.75 mm filament. The resistance values obtained for each filament are summarised in Table 1. Both the 80:20 and 60:40 mixes show the lowest resistance; whereafter, increasing the amount of graphite within the filament causes a sharp increase. This is attributed to the morphologies of the nanocarbons themselves, whereby the carbon black is present as small spherical particles and the graphite is present as large flakes, as seen in the SEM images of the powders in Figure S2. It is thought that the combination of small amounts of graphite flakes in combination with a larger number of small particles improves the electrically conductive network through the polymer, enhancing the conductivity. Once the concentration of large flakes becomes too high, there is inadequate linking between the dispersed graphite to form a good conductive network.

**Table 1:** Optimisation of the carbon black (CB) and graphite (G) loading within recycled PLA additive manufacturing filament at an overall loading of 15 wt%. Summarising the resistance across 10 cm of filament, the reduction peak current (*I*_*p*.*a*._) at 25 mV s^−1^, the heterogeneous electron (charge) transfer rate constant (*k*^0^), and the electrochemical active area (*A*_*e*_)

CB to G ratio	Resistance (kΩ)	*−I*_*p*.*a*._ (µA)	* k*^0^ (× 10^−4^ cm s^−1^)	*A*_*e*_ (cm^2^)
100:0	20 ± 1	22 ± 3	0.4 ± 0.2	0.14 ± 0.02
80:20	6.5 ± 0.5	49 ± 9	3 ± 2	0.35 ± 0.05
60:40	6.9 ± 0.7	49 ± 3	3.1 ± 0.4	0.32 ± 0.04
40:60	18 ± 2	27 ± 3	0.3 ± 0.1	0.19 ± 0.02
20:80	5500 ± 400	N/A	N/A	N/A
0:100	N/A	N/A	N/A	N/A

Next, the electrochemical performance of additive manufactured electrodes printed from these filaments was analysed using scan rate studies against the near-ideal outer sphere redox probe [Ru(NH_3_)_6_]^3+^ (1 mM in 0.1 M KCl). This probe is chosen as it allows for the best determination of the heterogeneous electron (charge) transfer rate constant (*k*^0^) and the real electrochemical surface area (*A*_*e*_) of the additive manufactured electrodes [38]. A summary of the findings can also be found in Table 1. It can be seen that again, the filaments comprised of 80:20 and 60:40 CB to graphite ratio produced the best results, with a faster *k*^0^ and larger *A*_*e*_, showing good agreement with the data obtained for the filament resistances. To maximise both the environmental and cost benefits of the filament, the larger the proportion of graphite within the filament, the better. This is due to the fact that carbon black is produced using the partial oxidation of petrochemical precursors, whereas graphite can be obtained from naturally occurring sources. As such, the ratio of 60 wt% CB and 40 wt% graphite was chosen to progress with.

### Physicochemical characterisation of optimised filament

**Fig. 1:**
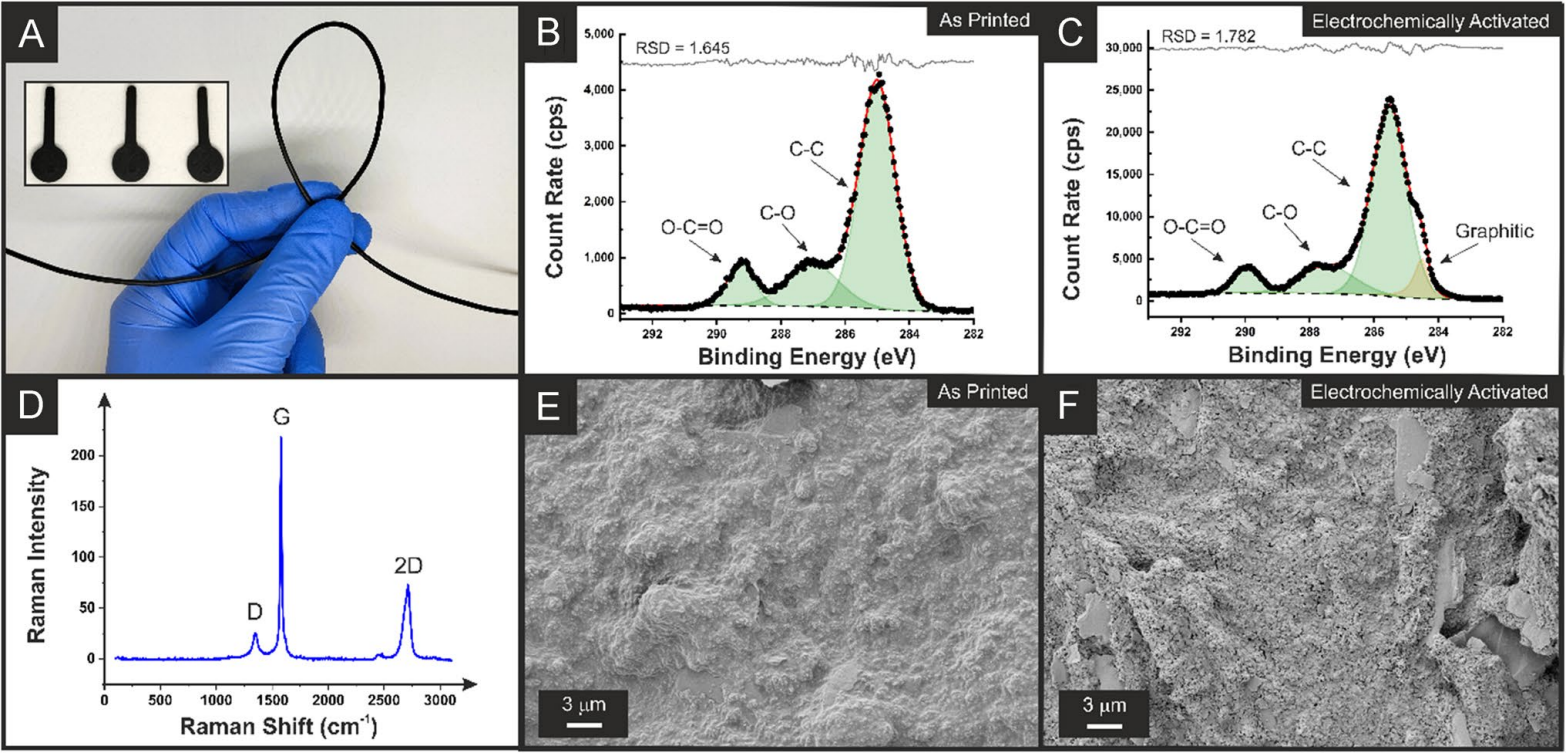
**A** Photograph of the bespoke filament comprised of 55 wt% recycled PLA, 10 wt% castor oil, 21 wt% carbon black, and 14 wt% graphite. Inset is a picture of three additive manufactured electrodes printed from this filament. XPS C 1s spectra for the **B** as-printed and **C** activated additive manufactured electrodes printed from the bespoke carbon black and graphite filament. **D** Raman spectra for the activated electrode. SEM micrographs for the surface of the **E** as-printed and **F** activated additive manufactured electrodes

Utilising the optimal ratio of nanocarbons calculated above, the maximum loading of this mixture was then explored within the rPLA, once again maintaining a constant 10 wt% castor oil as the plasticiser [26]. Then, subsequent filaments prepared with 15, 20, 25, 30, 35, and 40 wt% of total carbon loading were thoroughly tested. It was found that a total of 35 wt% nanocarbon loading in this ratio was possible before the filament produced became significantly more brittle, leading to a reduction in printability. The filament obtained using 35 wt% total loading, comprising 21 wt% CB and 14 wt% graphite, is pictured in Fig. 1A, which demonstrates the excellent low-temperature flexibility of this filament. Across 10 cm of this filament, an extremely low average resistance of 277 ± 9 Ω was measured, which matches the published bespoke filament range from ~ 250 to 1000 Ω [26–28, 39] and represents a significant improvement on the commercially available conductive PLA which has a quoted resistance of 2–3 kΩ [40, 41]. Importantly, this filament achieves such a level of conductivity at a low material cost of under £0.06 per gram, compared to £0.72 per gram previously reported [27] and a current purchasing cost of commercial filament of ~£1.20 per gram.

It is interesting to see the inset of Fig. 1A corresponds to an image of three additive manufactured electrodes printed from this filament, highlighting the excellent printing quality, surface finish, and reproducibility.

Once printed, it is important to characterise the surface of the additive manufactured electrodes to gain insight into their performance. Within the field of additive manufactured electrochemistry, especially when utilising PLA-based materials, activation of the electrode surface is commonplace. This term effectively refers to the removal of an outer layer of polymer that coats the electrode surface, allowing access to increased amounts of conductive filler below. There have been numerous ways of activating additive manufactured electrodes reported within the literature [42, 43], with electrochemical activation in a sodium hydroxide solution (0.5 M) being one of the most popular [35], and therefore, it will be used throughout this work. Then, additive manufactured electrodes were analysed through X-ray photoelectron spectroscopy (XPS), Raman spectroscopy, and scanning electron microscopy (SEM) before and after electrochemical activation.

Figure 1B, C shows the XPS C 1s spectra for the as-printed and electrochemically activated additive manufactured electrodes, respectively. The as-printed spectra show excellent agreement with previously published C 1s spectra for additive manufactured electrodes that utilise rPLA as the base polymer and castor oil as the plasticiser [26]. To adequately fit the as-printed electrode, three symmetric peaks were fitted, with the C-C peak at 285.0 eV being significantly larger in magnitude. This indicates the presence of castor oil on the surface of the print, as PLA exhibits three symmetric peaks of similar intensities [30]. When activated (Fig. 1C), an additional peak is required for adequate fitting of the C 1s spectra. The addition of an asymmetric peak at 284.5 eV is consistent with the X-ray photoelectron emission of graphitic carbon [44, 45]. This provides evidence that the electrochemical activation has exposed significant amounts of nanocarbons, with the atomic concentration increasing from 0 in the as-printed sample to 7.85% in the activated sample. The removal of the non-conductive PLA and castor oil from the surface means that the nanocarbons are now within the range of the XPS (a few nanometers) and expose the edge plane/site defects at the triple phase boundary, which should lead to enhanced electrochemical performance toward inner-sphere redox molecules.

To further confirm the presence of the nanocarbons on the surface of the additive manufactured electrodes, Raman analysis was performed (Fig. 1D). There are clearly defined peaks present at 1338, 1572, and 2680 cm^−1^, which are attributed to the characteristic F-, G-, and 2D-bands found within the Raman spectra for graphitic-like structures. The *I*_*D*_/*I*_*G*_ ratio for these peaks was calculated to be 0.12, showing a low number of defects and a very ordered structure, which indicates the presence of graphite on the surface of the electrode. These findings are further supported by the SEM images for the as-printed and activated additive manufactured electrodes seen in Fig. 1E, F, respectively. For the as-printed electrode (Fig. 1E), the morphologies of the nanocarbons can be observed, but there is a clear covering of smooth material attributed to both the plastic polymer and castor oil. On the other hand, for the activated electrode (Fig. 1F), there is a clear removal of this layer, and the well-defined morphologies of the carbon black and graphite become apparent. This characterisation clearly provides evidence that the electrochemical activation removes PLA and castor oil from the surface of the additive manufactured electrode, exposing increased amounts of conductive nanocarbons below. This is expected to lead to an improved electrochemical performance of the electrodes.

### Electrochemical characterisation of the additive manufacture electrodes

**Fig. 2:**
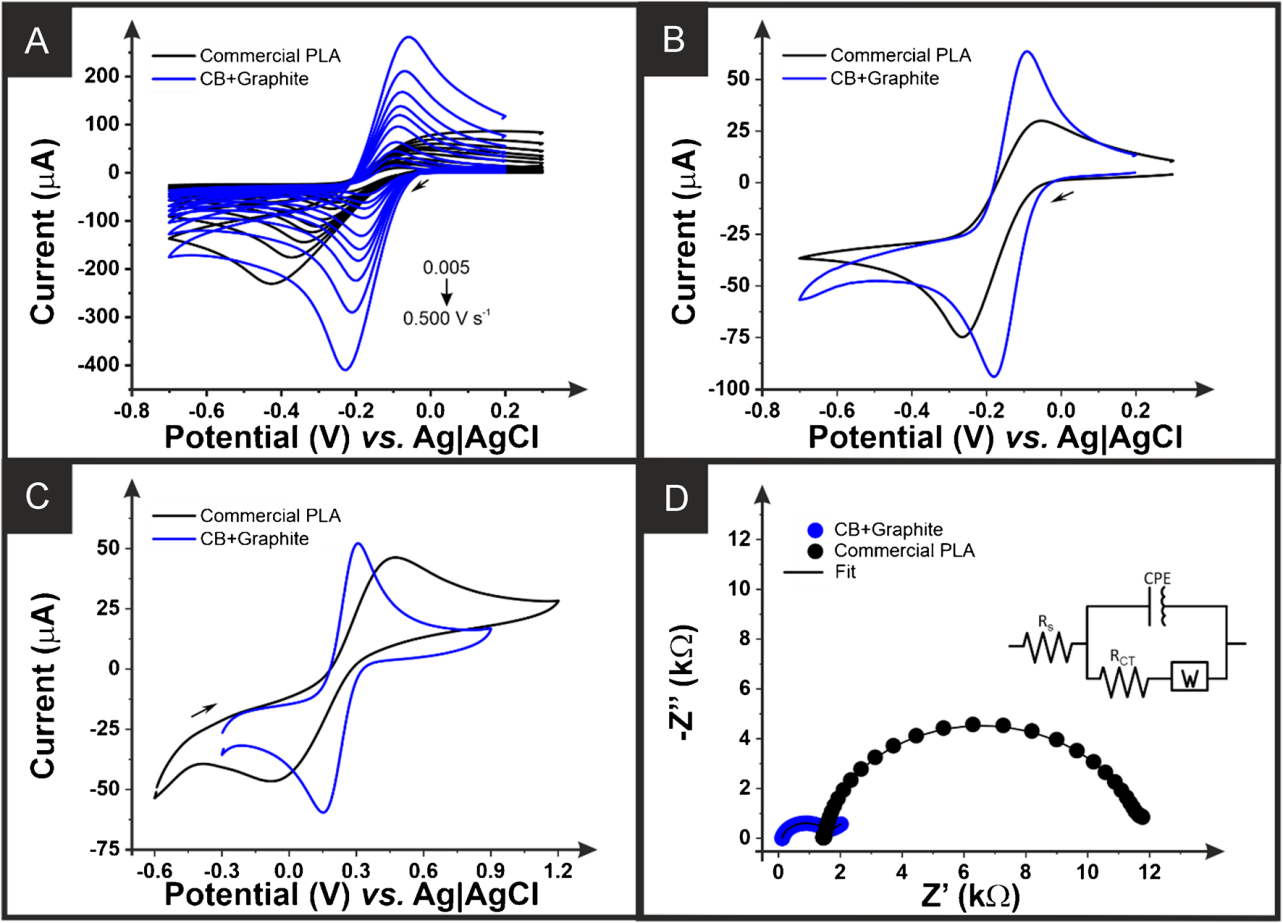
**A** Scan rate study (5–500 mV s^−1^) in [Ru(NH_3_)_6_]^3+^ (1 mM in 0.1 M KCl) performed with additive manufactured working electrodes printed from the CB-G/PLA filament (blue) and the commercial conductive PLA (black), nichrome coil counter electrode, and Ag|AgCl/KCl (3 M) reference electrode. **B** Cyclic voltammograms (25 mV s^−1^) in [Ru(NH_3_)_6_]^3+^ (1 mM in 0.1 M KCl) performed with additive manufactured working electrodes printed from the CB-G/PLA (blue) and the commercial conductive PLA (black), nichrome coil counter electrode, and Ag|AgCl/KCl (3 M) reference electrode. **C** Cyclic voltammograms (25 mV s^−1^) in [Fe(CN)_6_]^4−/3−^ (1 mM in 0.1 M KCl) performed with additive manufactured working electrodes printed from the CB-G/PLA filament (blue) and the commercial conductive PLA (black), nichrome coil counter electrode, and Ag|AgCl/KCl (3 M) reference electrode. **D** Electrochemical impedance Nyquist plot (100,000–0.1 Hz) obtained in [Fe(CN)_6_]^4−/3−^ (1 mM in 0.1 M KCl) performed with additive manufactured working electrodes printed from the bespoke CB-G/PLA filament (blue) and the commercial conductive PLA (black), nichrome coil counter electrode, and Ag|AgCl/KCl (3 M) reference electrode

Once physicochemically characterised, the additive manufactured electrodes were tested for their electrochemical performance. This was first done through scan rate studies against the near-ideal outer sphere redox probe [Ru(NH_3_)_6_]^3+^ (1 mM in 0.1 M KCl), as this allowed for the best determination of the heterogeneous electron (charge) transfer rate constant (*k*^0^) and the real electrochemical surface (*A*_*e*_) [38]. An example of the scan rate study (5–500 mV s^−1^) obtained for the bespoke filament and the commercial PLA used as a benchmark are presented in Fig. 2A, with only the scans obtained at 25 mV s^−1^ are presented in Fig. 2B. It can be observed that there is a large improvement in terms of both the measured peak currents and the peak-to-peak separations (*ΔE*_*p*_) when using the CB-G/PLA. At 25 mV s^−1^, the *ΔE*_*p*_ measured for the bespoke filament is 82 ± 4 mV compared to 212 ± 7 mV for the commercial conductive filament. This translates into a huge improvement in the *k*^0^, with the CB-G/PLA filament producing a value of (2.6 ± 0.1) × 10^−3^ cm s^−1^ compared to (0.46 ± 0.03) × 10^−3^ cm s^−1^ for the commercial PLA.

To further evaluate the electrochemical performance of the CB-G/PLA filament after being activated, it was tested against the inner-sphere redox probe [Fe(CN)_6_]^4−/3−^ (1 mM in 0.1 M KCl). Figure 2C presents the cyclic voltammograms (25 mV s^−1^) obtained using additive manufactured electrodes printed from the bespoke filament and commercial conductive PLA after activation. It can be seen that once again, there is a significant improvement in the *ΔE*_*p*_, with the CB-G/PLA filament producing a value of 135 ± 8 mV compared to 500 ± 34 mV for the commercial conductive PLA, indicating a massive improvement in electrochemical performance. Finally, the additive manufactured electrodes were tested through electrochemical impedance spectroscopy recorded between frequency values from 0.1 to 100,000 Hz against [Fe(CN)_6_]^4−/3−^ (1 mM in 0.1 M KCl). EIS allows for the accurate determination of the resistance introduced into the system by the additive manufactured electrode through the calculation of the solution resistance (*R*_*s*_) and the resistance to the electrochemical processes happening at the electrode/solution interface through the charge-transfer resistance (*R*_CT_) [46, 47]. The Nyquist plots obtained for additive manufactured electrodes printed from both bespoke and commercial PLA can be seen in Fig. 2D. There is clearly a significant disparity in both the *R*_*s*_ and *R*_CT_ values obtained for each electrode. The CB-G/PLA electrode produced an *R*_*s*_ and *R*_CT_ of 117 Ω and 1414 Ω, respectively, compared to 1466 Ω and 10,065 Ω for the commercial electrode. Since the *R*_CT_ parameter is inversely proportional to the heterogeneous electron (charge) transfer rate constant (*k*^0^) [48], the results obtained from EIS analysis corroborate that our bespoke graphite filament, displaying a significantly lower *R*_CT_ and higher *k*^0^ values, is a better candidate for enhanced electrochemical performance in further potential applications of this material.

### Electroanalytical determination of *β*-estradiol

**Fig. 3:**
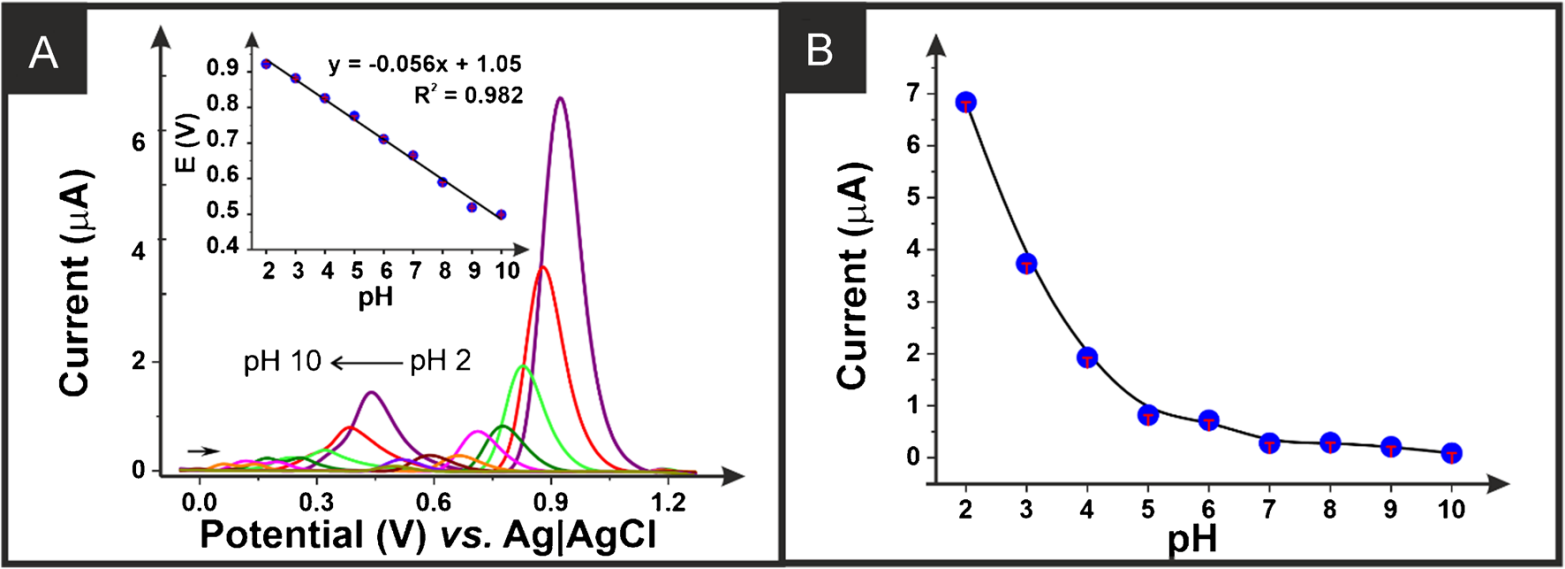
**A** Square wave voltammograms (amplitude: 20 mV; frequency: 25 Hz; step: 5 mV) for the detection of *β*-estradiol (100 µM) in 0.1 M Britton-Robinson buffer at different pH (2 to 10) performed with an additive manufactured working electrodes printed from the CB-G/PLA filament with a nichrome counter electrode and Ag|AgCl/KCl (3 M) reference electrode. Inset plot of the peak potential versus the pH for the data obtained in **A**. **B** Plot of the peak current versus the pH for the data obtained in **A**

Once electrochemically characterised, the additive manufactured electrodes printed from the CB-G/PLA filament were applied for the electroanalytical determination of *β*-estradiol. First, the response of *β*-estradiol (100 µM) was tested through square wave voltammetry within Britton-Robinson buffer at different pH values (2–10) (Fig. 3A). Through plotting the obtained peak potentials against the pH, we obtain a gradient of 56 mV per pH, which indicates that there is an equal number of electrons and protons involved in the redox reaction of *β*-estradiol. Next, cyclic voltammetry recorded at different scan rates (*ν*) was performed alongside the Laviron approach to estimate the number of electrons transferred in this reaction. It is stated by Laviron’s theory that the slope value obtained from plotting peak potential versus Log *ν* (Figure S3) corresponds to −2.3RT/αnF, where *R* is the gas constant (8.314 JK^−1^mol^−1^), *T* is the thermodynamic temperature (298.15 K), *α* is the transfer coefficient (*α* = 0.5 for irreversible processes), *F* is the Faraday constant (96,485 Cmol^−1^), and *n* is the number of electrons transferred. Considering the slope value of 0.05022 obtained from Figure S3, the value of *n* calculated was 2.3. This work shows good agreement with the mechanism described previously in the literature for the irreversible oxidation of *β*-estradiol involving two electrons and two protons [49].

It is interesting to note the two voltametric peaks observed for *β*-estradiol at different pH. Therefore, in agreement with the literature [49], the second anodic peak has been selected as representative of the voltametric behaviour of *β*-estradiol in further analyses. Figure 3B shows the peak current values measured for *β*-estradiol within the different pH Britton-Robinson buffer solutions, clearly showing a maximum peak current obtained at pH 2. Following this, the response within different electrolyte systems was explored through the detection of *β*-estradiol (100 µM) in pH 2 solutions (0.1 M) of H_2_SO_4_, HCl, and H_3_PO_4_ alongside the Britton-Robinson buffer. The square wave voltammograms obtained are presented in Figure S4A, which clearly shows an increased peak current obtained whilst using H_2_SO_4_. Interestingly, the inset in Figure S4A shows the need of facilitating the solubility of *β*-estradiol in H_2_SO_4_. This was achieved by adding 20% methanol to the solution, which avoids *β*-estradiol precipitating in the electrochemical cell. Therefore, we established that for the exploration of the electroanalytical response toward *β*-estradiol, a 0.1 M solution of H_2_SO_4_ with 20% methanol is used.

**Fig. 4:**
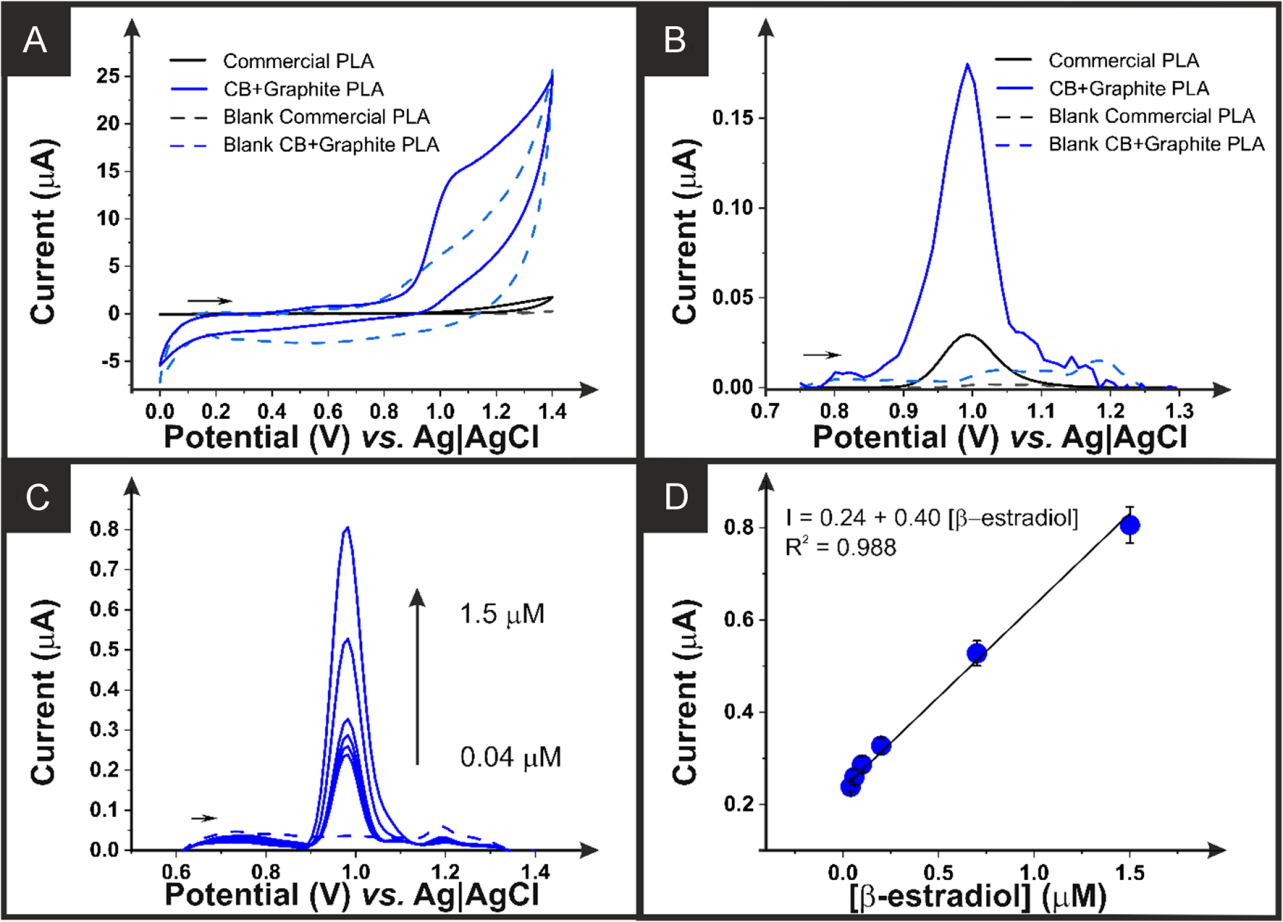
**A** Cyclic voltammograms (50 mV s^−1^) in the presence of *β*-estradiol (100 µM) in H_2_SO_4_ (0.1 M) with 20% methanol. Performed with additive manufactured working electrodes printed from the CB-G/PLA filament (blue) and the commercial conductive PLA (black) with a nichrome counter electrode and Ag|AgCl/KCl (3 M) reference electrode. **B** Square wave voltammograms in the presence of *β*-estradiol (1 µM) in H_2_SO_4_ (0.1 M) with 20% methanol. Performed with an amplitude of 10 mV, frequency of 5 Hz, and step potential of 10 mV, with additive manufactured working electrodes printed from the CB-G/PLA filament (blue) and the commercial conductive PLA (black) with a nichrome counter electrode and Ag|AgCl/KCl (3 M) reference electrode. **C** Square wave voltammograms in the presence of *β*-estradiol (0.04–1.5 µM) in H_2_SO_4_ (0.1 M) with 20% methanol. Performed with an amplitude of 10 mV, frequency of 5 Hz, and step potential of 10 mV, with additive manufactured working electrodes printed from the CB-G/PLA filament (blue) and the commercial conductive PLA (black) with a nichrome counter electrode and Ag|AgCl/KCl (3 M) reference electrode. **D** Calibration plot for the peak current data obtained in **C**

The cyclic voltammetric response of an additive manufactured electrode printed from the CB-G/PLA electrode and the commercial PLA within this matrix and in the presence of *β*-estradiol is presented in Fig. 4A. For the commercial PLA electrode, there is no peak observed for the oxidation of *β*-estradiol, whereas for the bespoke electrode, a clear irreversible peak is obtained at ~ 1.0 V vs. Ag|AgCl/KCl (3 M). For the electroanalytical detection of *β*-estradiol, more sensitive techniques than CV were tested, observing a better performance of SWASV compared to DPV, as shown in Figure S4B.

The square wave voltammetric responses of *β*-estradiol (1 µM) are shown in Fig. 4B for additive manufactured electrodes printed from the CB-G/PLA filament and the commercial PLA. It can clearly be seen that the CB-G/PLA electrode produces a significant improvement in the peak current values obtained, which were then further tested through the production of an electroanalytical curve. The square wave voltammograms for the detection of *β*-estradiol (0.04–1.5 µM) are presented in Fig. 4C, with the corresponding calibration plot in Fig. 4D. A linear response was found for the peak current over this concentration range, with the sensitivity calculated to be 400 nA µM^−1^, a limit of quantification (LOQ) of 70 nM, and a limit of detection (LOD) of 21 nM, highlighting the excellent performance of this electrode. Next, the electroanalytical performance of the additive manufactured electrodes printed from the bespoke carbon black and graphite filament was tested within real water samples to establish their real-world applicability.

**Fig. 5:**
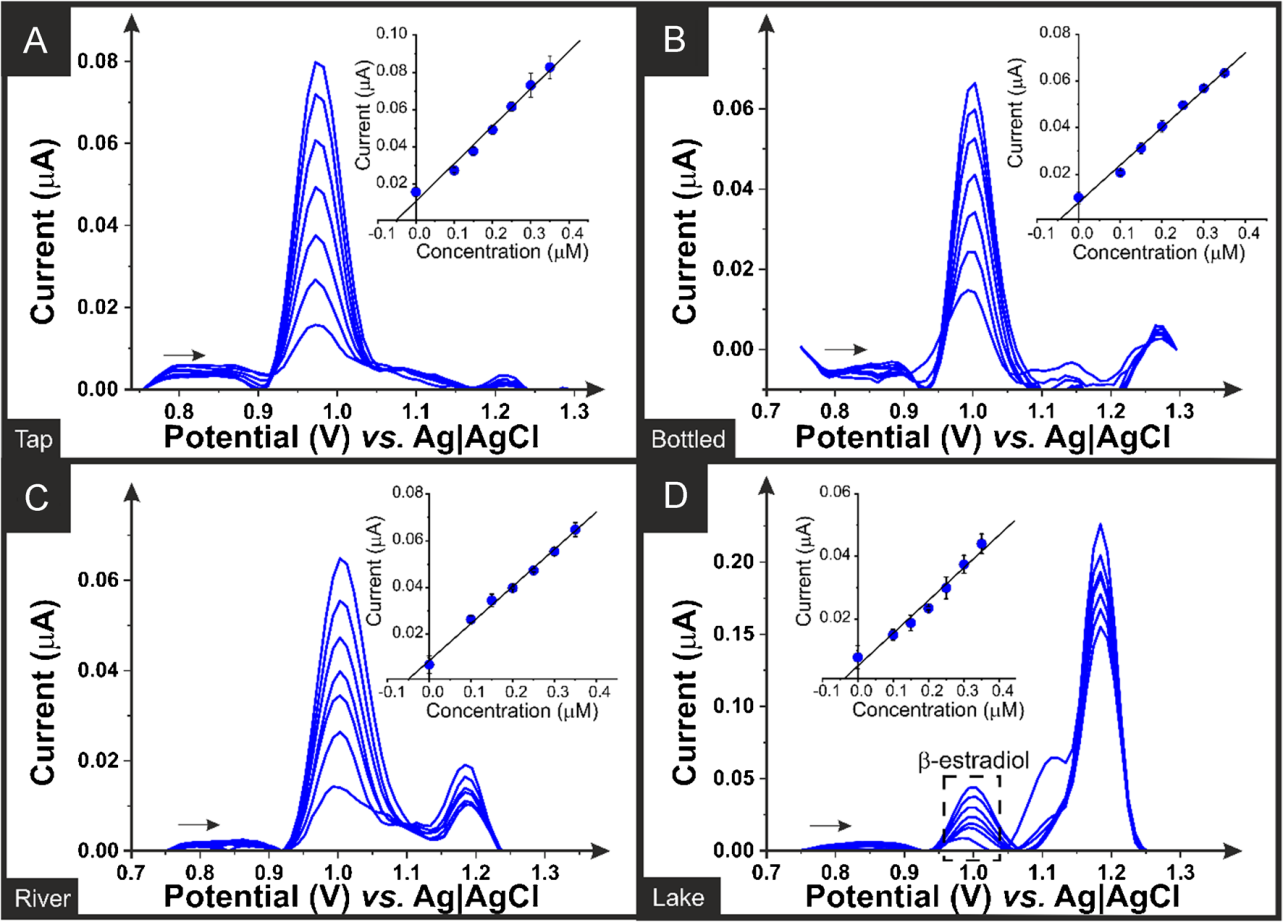
Square wave voltammograms and standard addition calibration plots (inset) for the determination of *β*-estradiol (0.1–0.4 µM) within **A** tap water, **B** bottled water, **C** river water, and **D** lake water samples. SWASV performed using an additive manufactured electrode printed from the bespoke CB-G/PLA filament, alongside a nichrome wire counter electrode and Ag|AgCl/KCl (3 M) reference electrode

The square wave voltammograms obtained for the determination of *β*-estradiol within four different real-world water samples are presented in Fig. 5. These included tap water obtained from the laboratory, bottled drinking water obtained from a local convenience store, and river and lake water obtained from local sources in accordance with EPA guidelines [50]. Note that tap and bottled water did not require any pretreatment, whilst river and lake water were filtered using a PTFE 0.45 μm syringe filter before the analysis to remove suspended natural particles. Quantification of *β*-estradiol in water samples was performed using the standard addition method, where successive additions of a standard solution of *ꞵ*-estradiol at concentrations of 100, 150, 200, 250, 300, 350, and 400 µM were added to the electrochemical cell. It is demonstrated that *β*-estradiol was successfully detected in all water samples. Interestingly, there is clearly an unknown contaminant within both the river and lake water samples, showing a large peak at ~ 1.2 V. Even so, the detection of *β*-estradiol was achieved, highlighting the excellent performance of these additive manufactured electrodes in real conditions.

The electroanalytical parameters obtained from the detection of *β*-estradiol within these water samples are summarised in Table 2, where they all show excellent results. The results obtained within the lake water sample show a reduction in sensitivity, LOQ, and LOD in comparison to the other samples which is attributed to the large contamination peak detected, which is clearly hindering the *β*-estradiol peak. Even so, excellent recovery values were obtained in all cases between 95 and 109%, showing evidence that this low-cost additive manufacturing filament provides excellent results toward quantifying *β*-estradiol in environmental samples and could be a great potential alternative for on-site analysis, considering both the simplicity of the methodology and the low cost of the electrode’s production (< £0.01).

**Table 2:** Analytical parameters obtained using the carbon black and graphite bespoke filament for the determination of *β*-estradiol in different water samples (*n* = 3), including the sensitivity, intercept, *R*^2^ value, limit of quantification (LOQ), limit of detection (LOD), and the recovery

Parameter	Tap water	Bottled water	River water	Lake water
Sensitivity (nA µM^−1^ )	201 ± 4	160 ± 7	159 ± 5	105 ± 8
Intercept (nA)	11 ± 2	8 ± 1	9 ± 1	5 ± 2
*R*^2^	0.984	0.991	0.994	0.973
LOQ (nM)	199	200	195	276
LOD (nM)	60	59	58	83
Recovery (%)	103 ± 1	100 ± 1	109 ± 1	95 ± 1

In comparison of the performance of the additive manufactured electrodes to other reports of *β*-estradiol within the literature (Table 3), it can be recognised that these electrodes perform exceptionally well. The obtained linear range and LOD compare to the literature, especially when considering the electrode materials and modifications required for some of the reports, with glassy carbon electrodes costing significantly more than the £0.01 per electrode from this filament. When compared to the only other additive manufactured electrode reported in the literature, it can be seen that the bespoke carbon black and graphite filament shows significant improvements in both the linear range and the LOD, improving this from 310 with the commercial PLA to 21 nM in this work.

**Table 3:** Comparisons between the electrode in this work and the literature for *β*-estradiol detection, including the electrode type used, electroanalytical technique, the real samples used, the obtained linear range, and the limit of detection (LOD)

Electrode	Technique	Sample	Linear range (µM)	LOD (nM)	Ref
GC/rGO/CuTthP	DPV	River water	0.01–1.0	5.4	[4]
Fe_3_O_4_-MIP/SPE	SWV	River water	0.05–10	20	[51]
CDs-PANI/GC	LSV	Drinking, tap, river water, & serum	0.001–100	43	[52]
Commercial PLA	DPV	Urine	2.5–75	310	[53]
rGO-AuNPs/CNT/SPE	DPV	Drinking water	0.05–1.0	3	[54]
Pt/Se/RDGC	CA	Tap & river water	0.05–85.5	11.12	[55]
CB/graphite	SWV	Tap, bottled, river, & lake water	0.04–1.5	21	This work
*GC/RGO/CuTthP*, glassy carbon modified with reduced graphene oxide and Cu(II)-meso-tetra(thien-2-yl)porphyrin; *Fe3O4-MIP/SPCE*, screen-printed carbon electrode modified with molecularly imprinted polymer based on magnetite nanoparticles; *CDs-PANI/GCE*, polyaniline/carbon dot-coated glassy carbon electrode; *3D-printed electrodes*, chemical treatment with DMF and electrochemical treatment with NaOH; *RGO-AuNPs/CNT/SPEs*, screen-printed carbon electrode based on gold-nanoparticle-decorated reduced graphene oxide–carbon nanotubes; *Pt/Se/RDGCE*, Pt nanoparticle-decorated Se rods-modified rotating-disc GCE (RDGCE)					

**Fig. 6:**
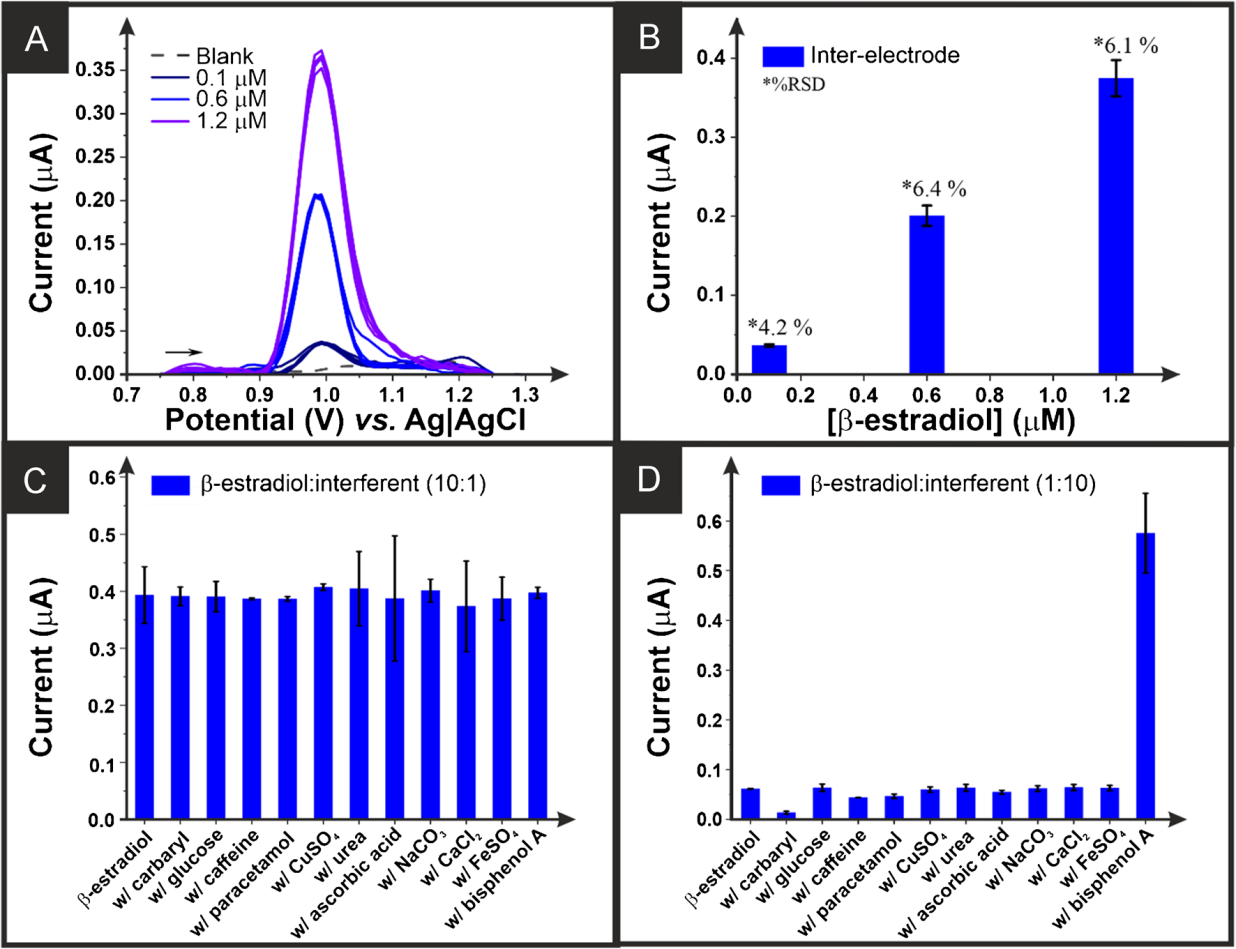
**A** Square wave voltammograms (amplitude: 10 mV; frequency: 5 Hz; step: 10 mV) for repeat measurements (*n* = 5) for the detection of *β*-estradiol (0.1, 0.6, and 1.2 µM). **B** Plot of the average peak current data obtained in **A**. Response of *β*-estradiol in the absence and presence of different interferents at a ratio of **C** 10:1 *β*-estradiol to interferent and **D** 1:10 *β*-estradiol to interferent

Finally, the reproducibility of the *β*-estradiol voltammetric signals and the interference study in the presence of other compounds of interest using the bespoke additive manufactured electrodes were studied. Figure 6A shows the square wave voltammograms obtained for the detection of three different concentrations of *β*-estradiol with five different electrodes. The average peak currents obtained are summarised in Fig. 6B, showing the excellent inter-electrode reproducibility of these additive manufactured electrodes. This can be attributed to the excellent low-temperature flexibility and printability obtained with this CB-G/PLA formulation.

The effect of eleven different possible interferents was then tested, and no interference was seen when the *β*-estradiol was present in a 10:1 excess (Fig. 6C). Moreover, when the molecules were present with a 10:1 excess of the interferent, there was no significant interference detected for caffeine, acetaminophen, copper sulphate, urea, ascorbic acid, sodium carbonate, calcium chloride, or iron sulphate. However, there were noticeable interferences at these concentration levels by carbaryl, which reduced the peak current by 78%, and by bisphenol A, which increased the peak current by 836% (Fig. 6D). This shows that for the accurate determination of *β*-estradiol in environmental samples potentially contaminated with those molecules, some matrix modification methods must be developed or utilised to ensure the reliability of the results obtained. Likewise, complementing the electroanalytical approach with validation using standard benchtop instrumentation, such as chromatographic methods, in those analyses where interferents could compromise the electrochemical determination of *β*-estradiol to give a high reading has to be considered to verify on-site results. Even so, the bespoke electrochemical platform developed in this work presents an extremely low cost, and it can be used as a portable method for the sensitive electroanalytical detection of low concentrations of *β*-estradiol within water samples. Through the creation of bespoke, low-cost additive manufacturing feedstock, there is the potential to revolutionise the ability to perform quick and simple field analysis.

## Conclusions

In this work, we report the production of a low-cost, electrically conductive additive manufacturing filament comprised of 55 wt% recycled PLA, 10 wt% castor oil, 21 wt% carbon black, and 14 wt% graphite. The ratio of carbon black to graphite was first optimised at lower loadings, where a ratio of 60% carbon black to 40% graphite produced the best compromise between electrochemical performance and material cost. Utilising this ratio, the maximum loading was then found to be an overall nanocarbon loading of 35 wt% alongside 10 wt% of the bio-based plasticiser castor oil within the PLA base polymer. This filament and the additive manufactured electrodes were physicochemically characterised through XPS, Raman, and SEM, showing that electrochemically activating the electrodes removed surface polymer, exposing significantly larger amounts of conductive carbons. These additive manufactured electrodes were electrochemically characterised against both [Ru(NH_3_)_6_]^3+^ and [Fe(CN)_6_]^3−/4−^, where they performed substantially better than the commercially available conductive PLA used throughout the literature.

The additive manufactured electrode was then applied toward the electroanalytical detection of *β*-estradiol within buffered solutions and real water samples, including tap, bottled, river, and lake water. In all cases, *β*-estradiol was successfully detected. In buffered solutions, the electroanalytical determination of *β*-estradiol achieved a sensitivity of 400 nA µM^−1^, a limit of quantification (LOQ) of 70 nM, and a limit of detection (LOD) of 21 nM, which compared remarkably to other reports in the literature. The detection within all four real samples was successfully achieved, obtaining recoveries between 95 and 109%. This work highlights how the creation of bespoke filament can massively increase the applicability of additive manufacturing electrochemistry for the electroanalytical detection of emergent environmental contaminants. Due to the ability to create high-performance filament at a low material cost (£0.06 per gram) and using more sustainable materials such as recycled polymers, bio-based plasticisers, and naturally occurring graphite, additive manufacturing will have a permanent place within the electroanalysis arsenal in the future.

### Electronic supplementary material

Below is the link to the electronic supplementary material.

Supplementary File 1 (DOCX 2.66 MB)
