# Supplementary material for: Optimised graphite/carbon black loading of recycled PLA for the production of low-cost conductive filament and its application to the detection of β-estradiol in environmental samples
Source: Mikrochim Acta. 2024 Jun 7;191(7):375. doi: 10.1007/s00604-024-06445-7 (PMC11161437; doi:10.1007/s00604-024-06445-7)
Supplement: Supplementary file 1 — (DOCX 2.66 MB) [file 604_2024_6445_MOESM1_ESM.docx]

**Supporting Information for:**

**Optimised graphite/carbon black loading of recycled PLA for the production of low-cost conductive filament and its application to the detection of β-estradiol in environmental samples**

Karen K. L. Augusto,^1,2^ Robert D. Crapnell,^1^ Elena Bernalte,^1^ Sabri Zighed,^1,3^ Anbuchselvan Ehamparanathan,^1,3^ Jessica L. Pimlott,^1^ Hayley G. Andrews,^1^ Matthew J. Whittingham,^1^ Samuel J. Rowley-Neale,^1^ Orlando Fatibello-Filho,^2^ and Craig E. Banks^1*^

*^1^Faculty of Science and Engineering, Manchester Metropolitan University, Chester Street,*

*M1 5GD, United Kingdom.*

*^2^Laboratório de Analítica, Bioanalítica, Biosensores, Electroanalítica e Sensores, Departamento de Química, Universidade Federal de São Carlos (UFSCar), CP 676, 13560-970 São Carlos-SP, Brazil.*

*^3^ Department of Physical Measurements, Sorbonne Paris North University, Place du 8 Mai 1945, 93200, Saint-Denis, France.*

^*^To whom correspondence should be addressed.

E-mail: [c.banks@mmu.ac.uk](mailto:c.banks@mmu.ac.uk); Tel: +44(0)1612471196

**
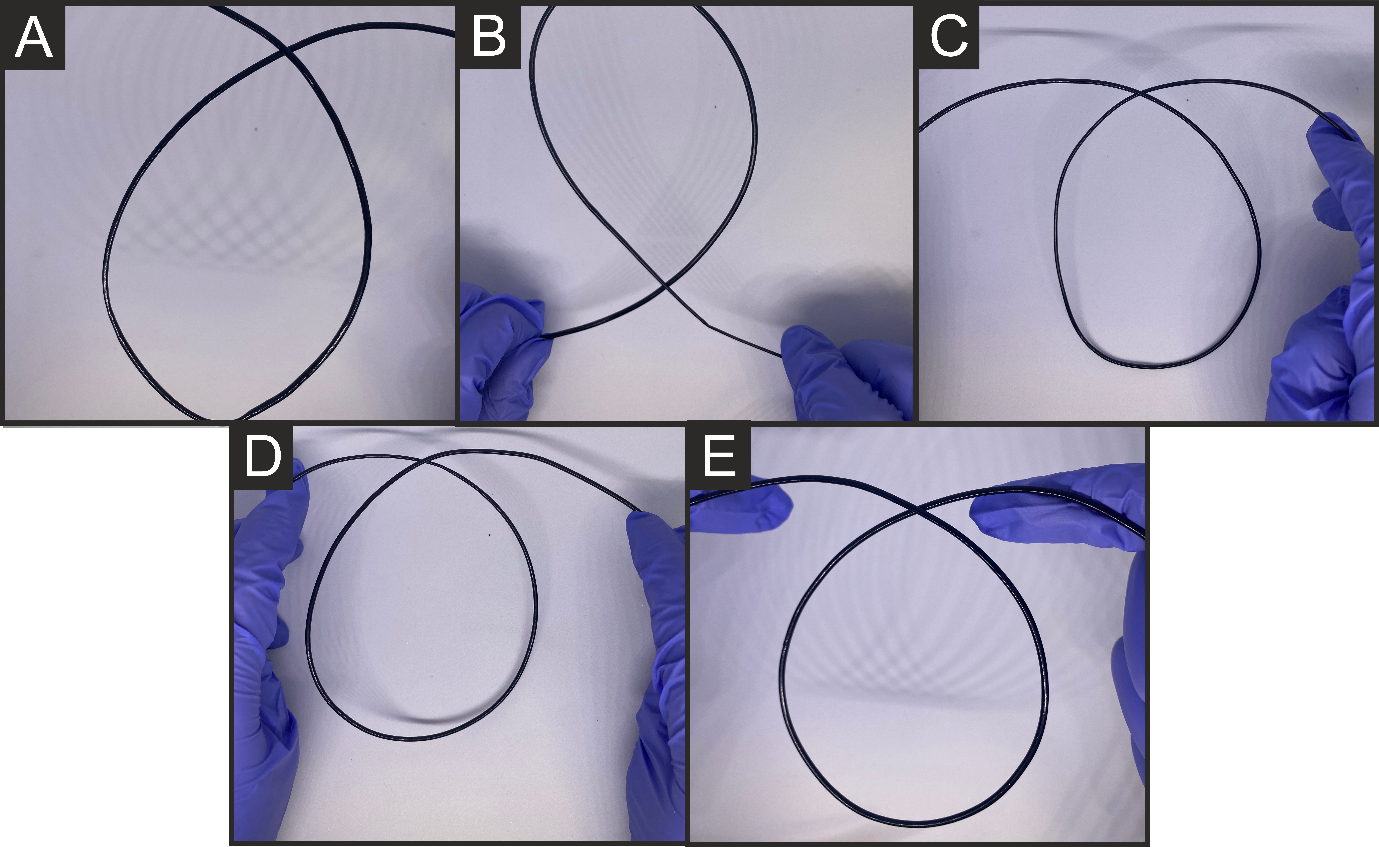
Figure S1.**  Photographs of 15 wt% filaments with different carbon black:graphite ratios: **A)** 100:0; **B)** 80:20; **C)** 60:40; **D)** 40:60 and; **E)** 20:80.


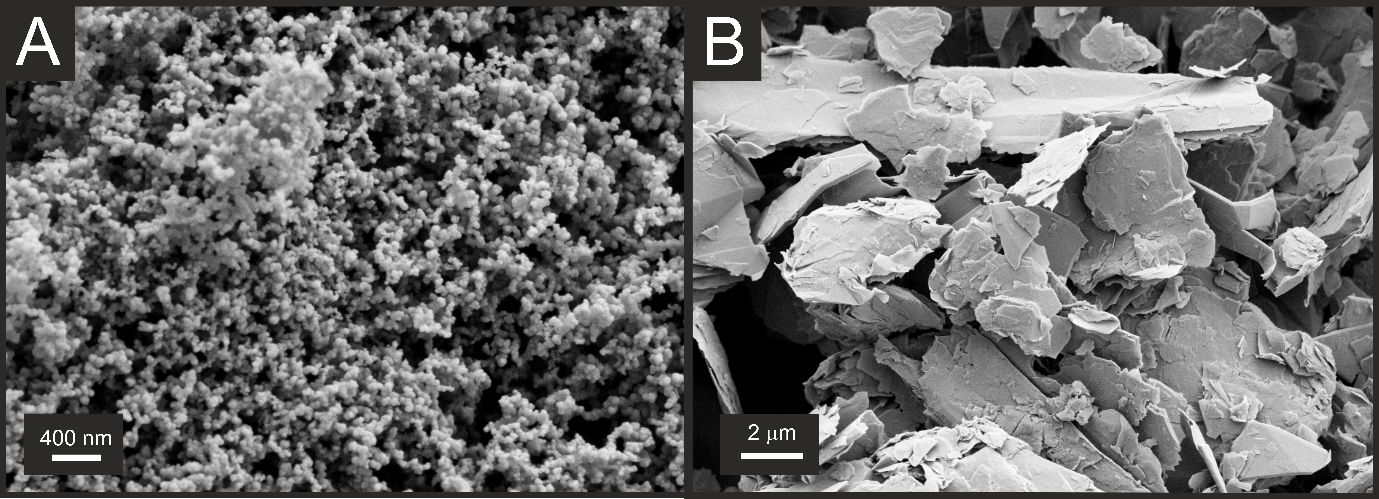


**Figure S2.** SEM micrographs for the powder samples of **A)** carbon black and **B)** graphite.


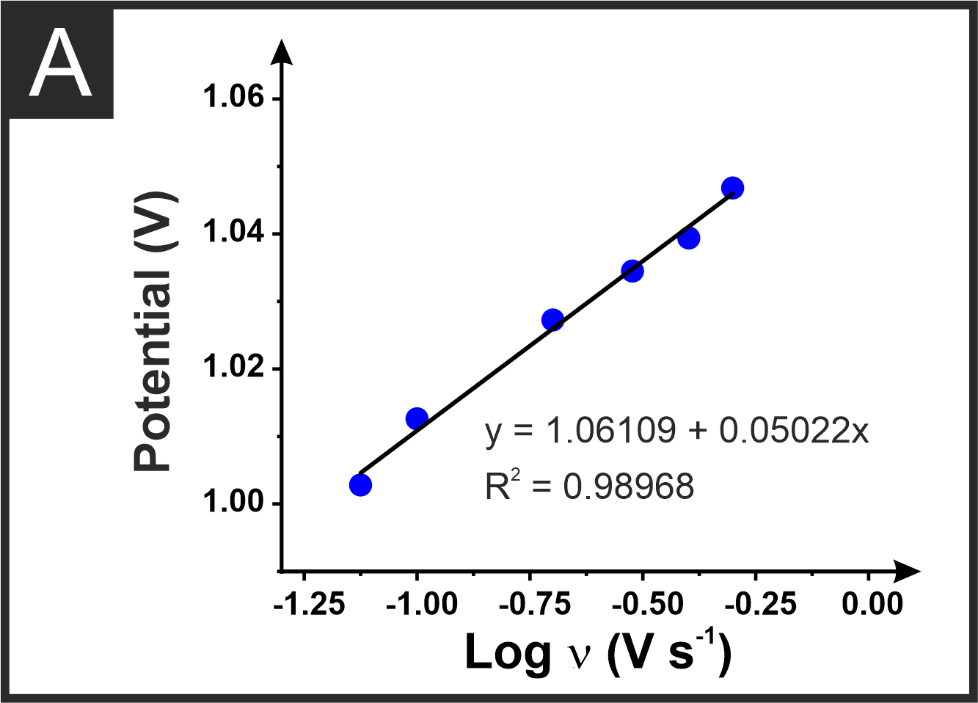


**Figure S3.** Plot of the peak potential versus the scan rate obtained from cyclic voltammograms (50 – 500 mV s^-1^) for β-estradiol (100 µM)


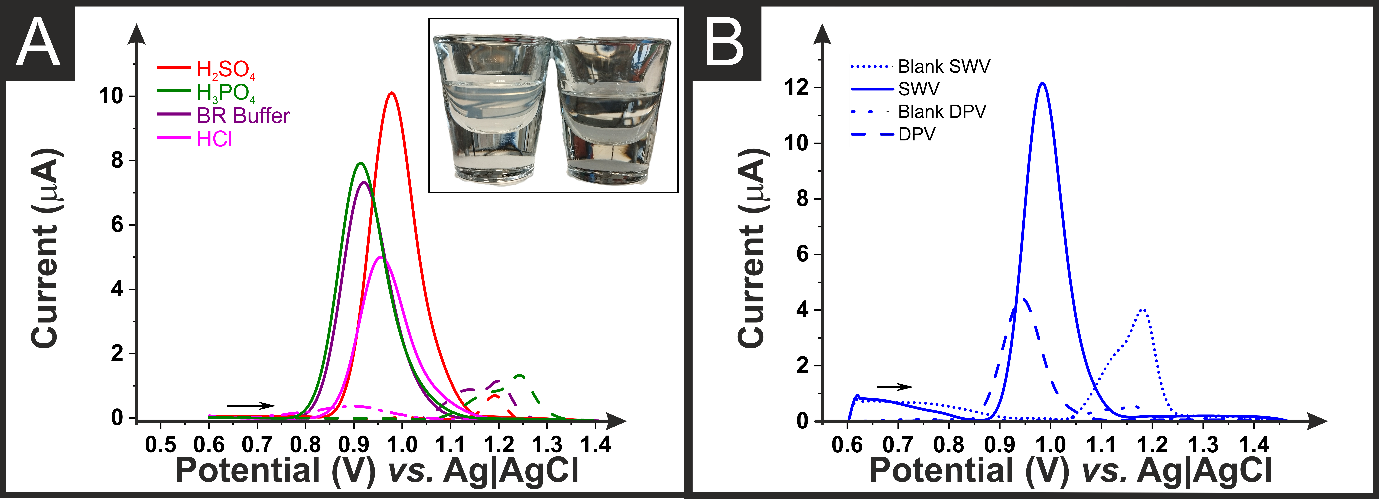
**Figure S4. A)** Square wave voltammograms for β-estradiol (100 µM) within different electrolytes (0.1 M). **B)** Comparison between the square wave and differential pulse voltammograms obtained for the detection of β-estradiol (100 µM) within H_2_SO_4_ (0.1 M) with 20% methanol.
